# Virus Capsid Dissolution Studied by Microsecond Molecular Dynamics Simulations

**DOI:** 10.1371/journal.pcbi.1002502

**Published:** 2012-05-10

**Authors:** Daniel S. D. Larsson, Lars Liljas, David van der Spoel

**Affiliations:** Department of Cell and Molecular Biology, Uppsala University, Uppsala, Sweden; Institut Pasteur, France

## Abstract

Dissolution of many plant viruses is thought to start with swelling of the capsid caused by calcium removal following infection, but no high-resolution structures of swollen capsids exist. Here we have used microsecond all-atom molecular simulations to describe the dynamics of the capsid of satellite tobacco necrosis virus with and without the 92 structural calcium ions. The capsid expanded 2.5% upon removal of the calcium, in good agreement with experimental estimates. The water permeability of the native capsid was similar to that of a phospholipid membrane, but the permeability increased 10-fold after removing the calcium, predominantly between the 2-fold and 3-fold related subunits. The two calcium binding sites close to the icosahedral 3-fold symmetry axis were pivotal in the expansion and capsid-opening process, while the binding site on the 5-fold axis changed little structurally. These findings suggest that the dissociation of the capsid is initiated at the 3-fold axis.

## Author Summary

We have studied the capsid of satellite tobacco necrosis virus using large scale molecular dynamics simulations, where the atomic motions of 1,2 million particles were tracked over one microsecond. We find that the capsid swells in the simulations, and that the permeability for water increases 10-fold upon removal of the structural calcium ions. The water leaks in predominantly near the three-fold symmetry axis, suggesting that this is the spot where capsid dissociation is initiated following infection.

## Introduction

Non-enveloped icosahedral viruses often contain binding sites for divalent cations, usually 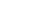. The ions are typically bound between coat proteins or on the icosahedral symmetry axes. This is broadly observed in three plant virus taxa: the family *Tombusviridae* (and an associate satellite virus), the genus Sobemoviruses and the family *Bromoviridae* [1]–[4]. Binding sites for calcium ions have also been found in bacteriophages of the *Leviviridae* family [5], fish and insect viruses of the *Nodaviridae* family [6] and in the *Picornaviridae* family, e.g. several human rhinoviruses [7].

In many of the plant viruses it is possible to induce a conformational change *in vitro* by removing the ions, either by a chelating agent such as ethylenediaminetetraacetic acid (EDTA) or by exhaustive dialysis against deionized water. Ion-deprived virions reversibly expand on the order of 5–10% at neutral or slightly alkaline pH. In the swollen state internal parts of the virion as well as the RNA molecule may become susceptible to degrading enzymes [8], [9]. Chelation of the metal ions is also required for synthesis of virus proteins in cell-free translation systems [9]. Only two low-resolution crystal structures of expanded virons are available: tobacco bushy stunt virus (TBSV) at 8 Å [10] and satellite tobacco necrosis virus (STNV) at 7.5 Å [11]. The radial increases are about 11% and 4%, respectively. In addition, an expanded cowpea chlorotic mottle virus (CCMV) virion was imaged with cryo-electron microscopy at 29 Å and interpreted using rigid body fitting of the high-resolution structures of the native proteins [4]. The dynamic nature of the swelling process as well as the limited resolution of swollen virus particles structures prompted us to perform a simulation study of the capsid of STNV, with and without bound 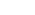, over one microsecond. The simulations allowed us to reproduce the swelling behavior upon removal of the calcium *in silico* and develop an atomistic description of the process.

The T = 1 capsid of STNV consists of 60 identical coat proteins with one protein per icosahedral asymmetric unit. The coat protein is 195 amino acid residues long where residues 25–195 make up the main domain that constitutes the capsid shell. The virions readily crystallize and the major part of the coat protein has been resolved by X-ray crystallography [2], [12]–[14]. The shell domain at the C-terminus folds as a 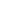-jelly roll similar to many other single-stranded RNA plant viruses. Residues 12–24 form a helical structure that together with the helices of two neighboring subunits form a short stalk that projects inwards into the central cavity around the icosahedral 3-fold axis. The first 11 residues at the N-terminus are disordered and cannot be detected in the electron density maps – in the simulations these residues were modeled as a helix as well. This N-terminal arm and the interior surface of the capsid are lined with positively charged residues that presumably interact with the single-stranded positive-sense RNA molecule [14]. The 1239 nucleotide long genome encompasses only one open reading frame that encodes the coat protein and hence STNV is dependent on the co-infection of a helper virus (tobacco necrosis virus) for copying its RNA genome.

**Figure 1:**
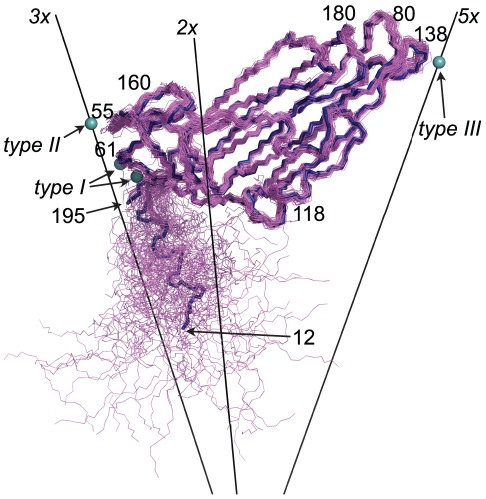
Fold of the coat protein. The main chain of all 60 proteins in 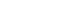 after 1 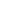 (violet) aligned to the crystal structure (blue). Symmetry axes and 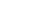 binding sites of all types are indicated, along with residues mentioned in the text.

The capsid has three different types of 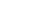 binding sites (Figure 1). Type I is between two subunits close to the 3-fold symmetry axis. The protein ligands are the carboxyl groups of Asp194 and Glu25 as well as the main chain carbonyl oxygens of Ser61 and Gln64. Type II is on the 3-fold symmetry axis 8.05 nm from the center of the virion. It is coordinated by the carboxyl groups of three Asp55 residues. Type III is on the 5-fold symmetry axis 9.04 nm from the center. This 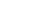 is coordinated by the main chain carbonyl oxygen of five Thr138 residues. In total the capsid can accommodate 92 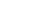 ions (60 at type I sites, 20 at type II sites and 12 at type III sites).

Simulations were performed of the capsid with and without 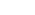 at two different salt concentrations for one microsecond each (Table 1). The carboxyl groups of one of the three Asp55 residues at each of the type II calcium binding sites were protonated in the two simulations without 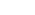, effectively simulating the capsid at a slightly acidic pH to mimic the conditions of the expanded capsid in the 7.5 Å crystal structure [11]. The RNA molecule was not included in our simulations since it cannot be modeled completely in the electron density maps [14], [15]. The aim of this work was to probe the dynamic behavior of a virus capsid over timescales that are more than an order of magnitude longer than what has been reported from simulations of viruses previously [16]–[18], and therewith to investigate the role of the structural calcium ions in initiating the dissolution of the satellite tobacco necrosis virus. We particularly looked into the structural features facilitating breaking up of the capsid associated with virus infection.

**Table 1:** System compositions and simulation results.

	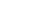	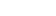	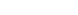	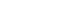
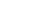	92	92	0	0
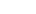	0	657	0	657
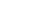	664	1321	500	1157
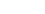	330540	329226	330704	329390
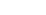	+0.78%	+0.58%	+2.6%	+2.4%
Crossings	13838	10204	150689	92090
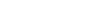	2.7 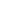 0.3	2.4 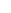 0.3	37.2 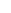 1.9	26.8 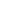 1.5
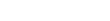	1.1 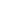 0.1	1.0 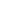 0.1	14.9 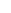 0.8	10.8 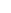 0.6
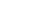	11.6 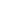 0.03	11.9 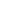 0.03	9.7 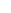 0.02	10.7 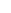 0.03
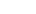	8.2 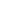 0.7	8.7 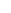 0.6	6.5 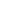 0.5	7.2 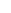 0.5
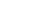	2.8 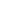 0.1	3.0 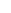 0.2	1.7 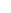 0.1	1.5 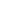 0.1
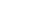	10.2 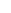 0.1	10.1 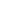 0.1	10.1 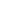 0.2	10.0 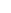 0.2
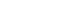 (nm)	0.11 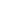 0.02	0.10 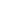 0.01	0.15 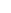 0.02	0.15 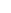 0.03
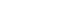 (nm)	0.13 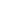 0.01	0.13 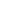 0.01	0.20 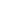 0.02	0.20 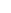 0.02
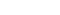 (nm)	0.11 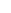 0.01	0.11 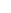 0.01	0.15 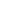 0.03	0.15 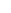 0.03

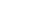
: Increase in the radius of gyration (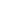 of res. 25–195). Crossings: Water permeation events during 1 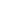. 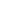: Osmotic water permeability coefficient. 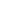: Single pore osmotic water permeability coefficient for the 3-fold axis. 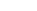: Number of 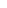-helix residues in the N-terminus. 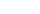: Number of intermolecular hydrogen bonds between pairs of subunits related by 2-, 3- or 5-fold symmetry (disregarding interactions between the N-terminal 11 residues). 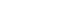: RMSd from the crystal structure for 2-, 3- or 5-protein oligomers calculated for the main domain (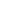 of residue 25–195). Average values from the last 0.1 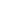 where the ranges indicate the standard error.

## Results

### The capsid expands upon calcium removal

**Figure 2:**
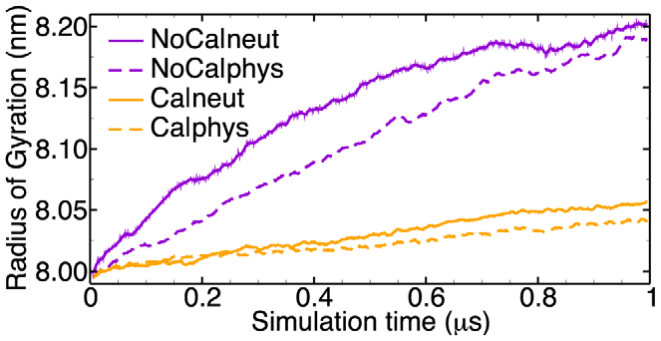
Radius of gyration. The 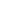 of the 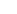 atoms of the shell domain (residues 25–195) plotted every 1 ns with a running average over 10 points (non-averaged data also shown for 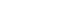).

The capsids were stable and remained intact throughout all four trajectories. Removing the 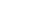 had a pronouced effect on the capsid radius. An increase in the radius of gyration (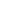) of 2.6% in 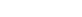 and of 2.4% in 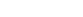 was found over the course of 1 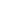. The two trajectories with bound 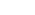 (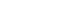 and 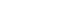) showed a weak tendency to increase in size, 0.78% and 0.58% respectively (Table 1 and Figure 2).

**Figure 3:**
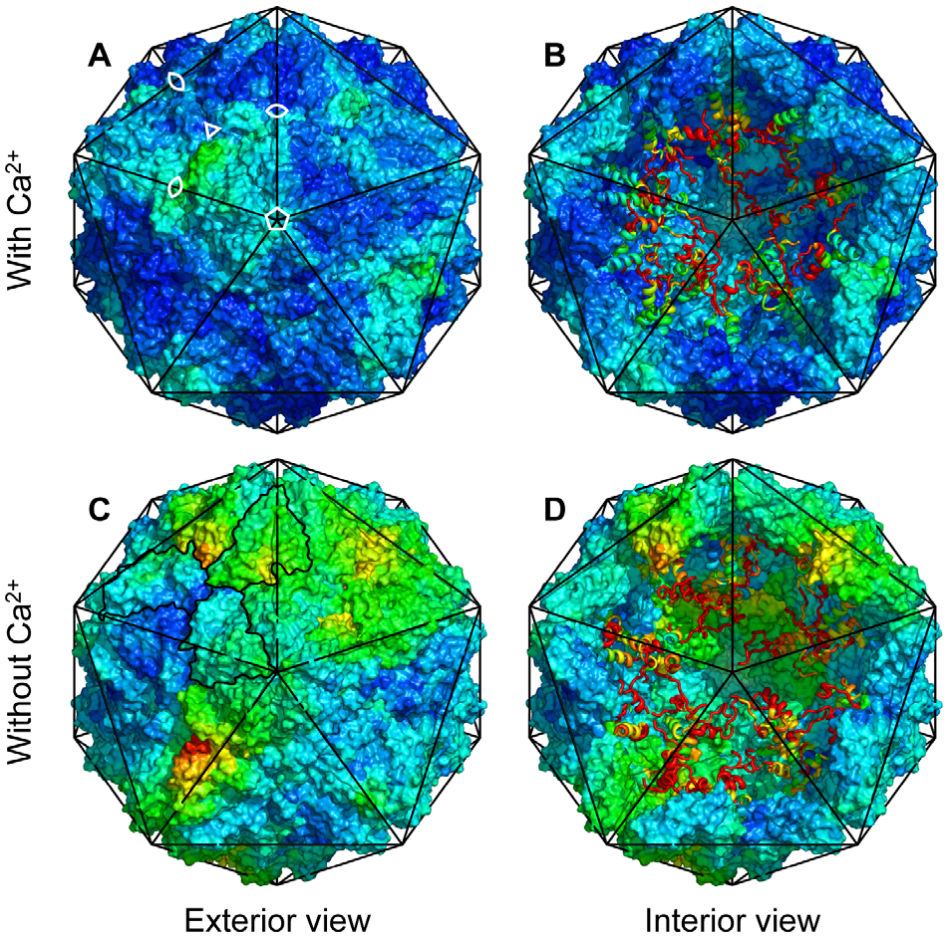
Structural changes of the capsid. Van der Waals surfaces of the capsid shell after 1 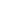 viewed along one of the 5-fold symmetry axes. Top row (**A, B**) shows 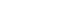 and bottom row (**C, D**) shows 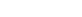. Right column (**B, D**) shows the interior structure when the 15 frontmost proteins has been removed. Each capsid inscribed in an icosahedron with 22 nm between opposing vertices. The N-terminal domains are represented as ribbons. Colors according to the RMSd from the crystal structure. The color scale goes from blue (0.0 nm) through cyan, green, lime-green, yellow and red (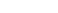). Location of symmetry axes marked for one icosahedral face in (**A**). The outline of the proteins in one trimer traced in (**C**).

**Figure 4:**
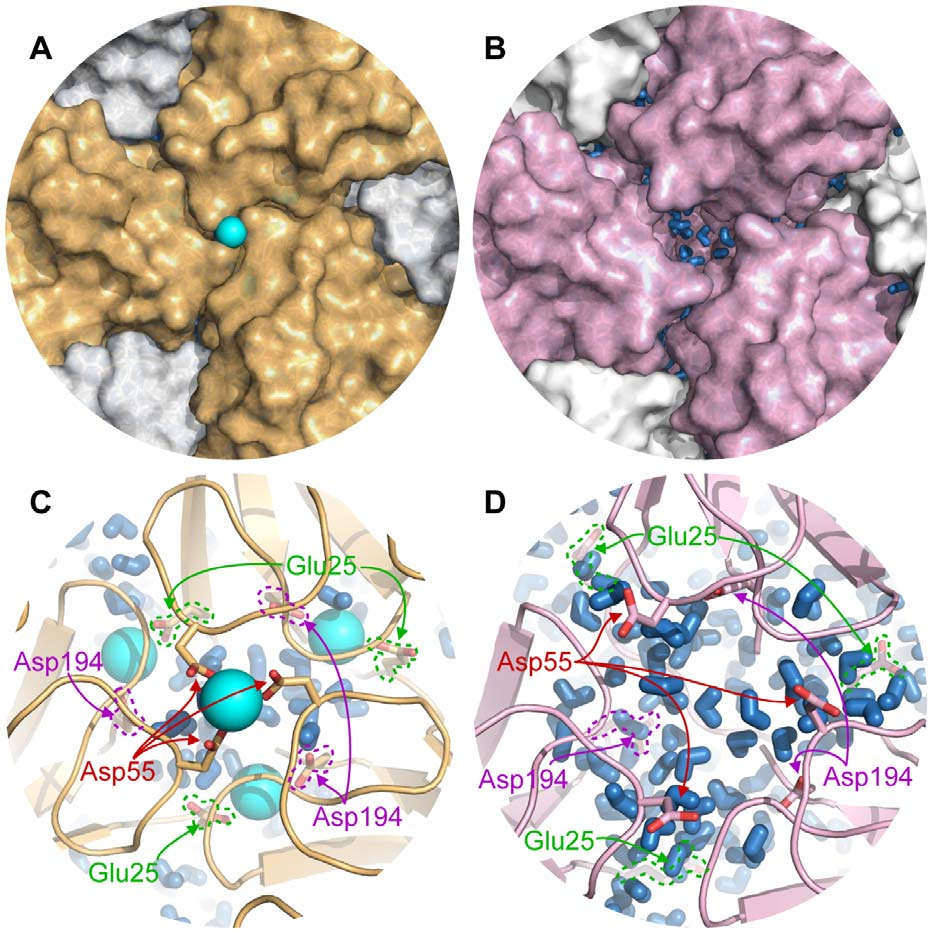
The 3-fold axis. Close-up of an icosahedral 3-fold symmetry axis from the final frame of the (**A, C**) 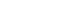 and the (**B, D**) 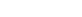 trajectories showing the main water permeation site. Calcium ion depicted as a cyan sphere and water molecules as blue sticks.

The capsids retained their overall spherical shape with just some minor degree of elongation. Figures 3A and 3C emphasize the local anisotropy in the structural changes. The largest changes occurred in the parts of the shell close to the icosahedral 3-fold axes. This area accommodates four calcium binding sites and all of them have charged carboxyl groups as ligands. The charge repulsion induced the formation of small water-filled cavities between the proteins at this protein/protein interface (Figure 4D). An analysis where the root mean square deviation (RMSd) from the crystal structure for protein dimers, trimers and pentamers was computed is presented in Table 1. The trimers have clearly larger than average RMSd, whereas dimers and pentamers form very stable complexes. This effect is more pronounced in the simulations without 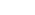.

### Dynamics of the coat protein

**Figure 5:**
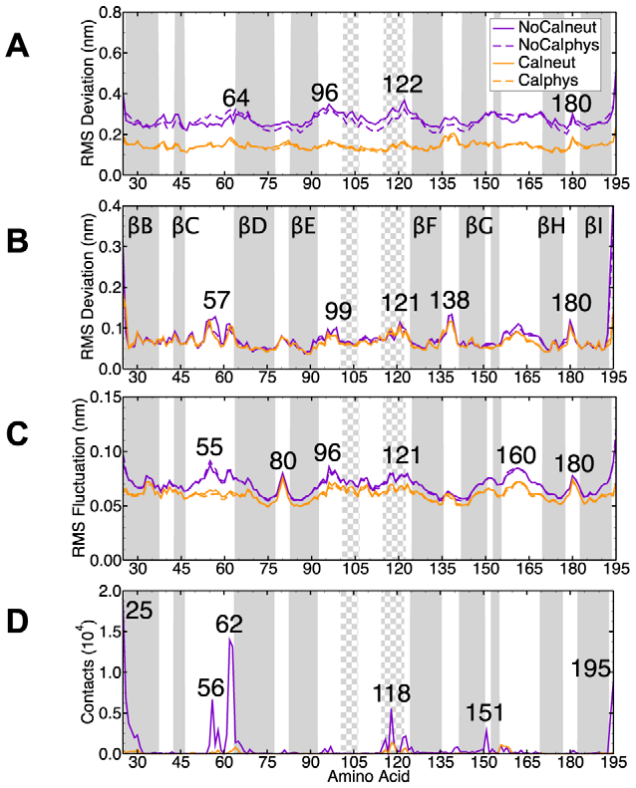
Structural dynamics of the capsid and water contacts. (**A**) The RMSd from the crystal structure (without over all rotation and translation of the entire capsid) for the 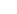 of the shell domain (average over the last 0.1 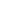 and all 60 proteins). (**B**) As **A** but after aligning each protein individually to the crystal structure. (**C**) The RMSf for the 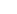 of the shell domain (average over the last 0.1 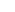). (**D**) The number of contacts to water molecules crossing the capsid shell. The secondary structure elements of the crystal structure are shown as gray (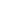-strand) or checkered (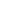-helix) regions.

The secondary structure elements and the overall fold of all the coat proteins were stable throughout the simulations. The N-terminal arm was the most flexible element (Figures 1, 3 and 5A). The 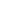-helix in the N-terminal arm observed in the crystal structure was stable: at the end of the simulation the number of 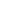-helical residues in the N-terminal arm was close to 11, slightly lower in the two trajectories without bound 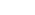 than the simulations with bound 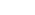 (Table 1). Residues 1–11 did not show any propensity to stay in a helical conformation. These residues were modeled as a helix in the starting structure, but they progressively lost that structure. This might be a result of the absence of the RNA molecule since the addition of molecules that mimic the phosphate backbone of RNA has been shown to promote formation of helices in the positively charged N-terminus of CCMV, that presumably plays a similar RNA-binding role [19], [20]. The number of intermolecular protein-protein hydrogen bonds was slightly lower in the STNV capsids without 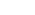, in particular the number of hydrogen bonds between pairs of 2-fold and 3-fold related subunits decreased (Table 1).

The 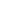 atoms in the shell domain moved on average 0.27 nm in 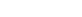, 0.26 nm in 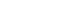, 0.15 in 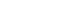 and 0.14 in 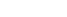 predominantly due to the overall radial expansion of the capsid (Figure 5A). The RMSd after fitting each protein to the crystal structure individually was small (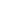
0.1 nm) apart from the termini and some of the loop regions (Figure 5B). The flexibility was highest in the two termini, in the loops between secondary structure elements and in the short helix centered at residue Thr119 (Figure 5C). The 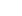
–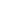 loop (using the same nomenclature as in [2]) centered at residue Thr80 and the 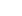
–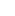 loop centered at residue Leu180 show high flexibility both with and without bound 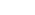 (Figure 5C). Both of these loops are located close to the 5-fold axis, facing the exterior of the capsid (Figure 1). The 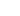
–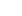 loop at residue Asp55, the 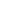
–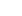 loop at residue Thr160 and the C-terminus on the other hand show an increased flexibility mainly in the two capsids that had no 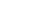 (Figure 5C). These elements are all located close to the 3-fold axis and Asp55 is the 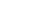 ligand for the type II sites.

### Calcium binding sites

When the 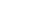 ions were removed from the capsid, the type III calcium binding sites on the icosahedral 5-fold symmetry axis were populated by either 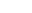 (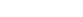 only) or water. 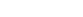 had 7 out of 12 type III binding sites occupied by sodium ions throughout the simulation. The remaining type III sites as well as all the type III sites in the capsid of 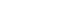 were occupied by one or two water molecules. The sodium ions were bound at the same position as the calcium ions – at the geometric center of the five Thr138 carbonyl groups – but half of the time, one of the five carbonyl groups pointed away from the 5-fold axis and engaged in hydrogen bonds with other water molecules. Water molecules bound to the type III sites were located on the 5-fold axis slightly exterior or interior to the binding site for cations such that they could make hydrogen bonding interactions with two or three carbonyl groups. In these cases the 5-fold symmetry of the protein subunits was broken with one (when two water molecules were bound) or two (when one water molecule was bound) carbonyl groups turned away and engaged in hydrogen bonds with water molecules from the bulk.

The two types of calcium binding sites close to the icosahedral 3-fold symmetry axis were less stable than the type III sites upon 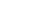 removal as reflected in the higher RMSd and RMSf of residues close to the 3-fold axis (Figure 5). In 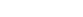 all type II sites were occupied by one or two sodium ions subsequent to the equilibration simulation (in which the protein was restrained from moving away from the crystal structure). However, at the end of the production simulation only 1 of the 20 sites was intact with all three carboxyl groups of Asp55 coordinating a sodium ion. The rest of the type II sites were broken up with the carboxyl groups facing other directions(Figure 4D). The type I site also lost its structure in the calcium-free capsids. The four residues that contributed oxygen atoms to coordinate the calcium ion had high RMSf (Figure 5C) and did not retain their relative positions. The negatively charged carboxyl side chains of all the type I sites together bound approximately 30 sodium ions in an irregular fashion.

### Water permeability

The capsids were permeable to water in all trajectories. Assuming a homogeneous permeability across the entire surface of the capsid, an osmotic water permeability coefficient, 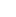, was calculated [21]. With 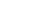 bound there were about 10,000 permeation events in either direction and the capsid had an average permeability coefficient 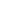 = 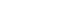 cm/s. After removing the 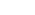 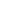 increased to about 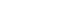 cm/s (Table 1). The water transport resulted in a net inflow of water in conjunction with the swelling.

**Figure 6:**
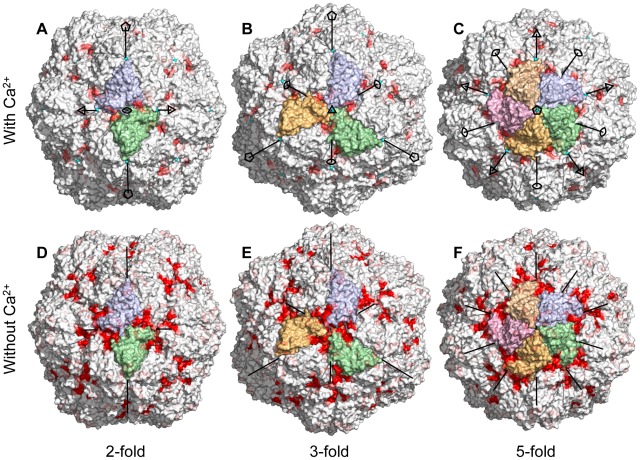
Water permeability mapped on the capsid surface. Van der Waals surface of the capsid after 1 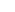 simulation. Red color indicates where water molecules pass in or out of the capsid (averaged over the icosahedral symmetry). Top row (**A–C**) with calcium is from 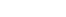 (calcium ions shown as cyan spheres). Bottom row (**D–F**) is from 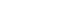. Views along an icosahedral 2-fold (**A, D**), 3-fold (**B, E**) and 5-fold (**C, F**) symmetry axis respectively. Subunits around the central symmetry axis colored for clarity.

In order to detect structural features associated with water permeability, the crossing events were mapped onto the virus particle. For each water molecule traversing the width of the capsid shell successfully, the closest protein residue (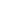) was determined for the water molecule half-way (in time) through, and statistics of permeation per residue were gathered. In the simulations with bound 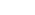 the permeation mainly occurred between the icosahedral 2-fold and 3-fold symmetry axis, at the junction between three subunits (Figures 6A–C and 4). The protein–water contacts suggest that this potential water pore is lined by five motifs: the short helix centered at residues 118–119, residues 156–158 in the 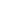
–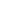 loop, the end of the 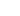
–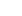 loop, the beginning of the 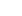-strand and to some degree residues 25–30 close to the flexible loop connecting the N-terminal arm to the shell domain (Figure 5D). In the simulations without bound 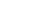 the permeability increased at this site, as well as at the protein/protein interface at the 3-fold symmetry axis (Figures 5D, 6D–F).

If we consider the region around each icosahedral 3-fold axis to be a water pore, we can estimate the single pore permeability coefficient, 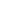, to be 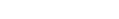 in the capsid with bound 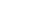 and 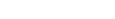 without these ions (Table 1).

## Discussion

Many plant viruses employ the extremely low concentration of 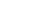 in the cytoplasm of their host cells (the homeostatic concentration of free 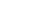 is between 100–200 nM [22]) as a cue to initiate the replication stage of their life-cycle. The capsid will be effectively depleted of calcium as the equilibrium between occupied and unoccupied sites shifts to the latter.

### Capsid swelling

Four different crystals of STNV treated with EDTA were investigated by Montelius *et al.*, but the capsid expanded in only one of those [11] and that particular crystal diffracted to a resolution of 7.5 Å only. In our simulations we could observe a higher degree of fluctuations as well as loss of the icosahedral symmetry in the calcium-deprived capsids compared to the ones with bound calcium. Both higher flexibilty and a lower degree of symmetry would contribute to a lower crystal quality and could explain why there have not been any successful attempts to solve a high-resolution structure of an expanded capsid so far. The trajectories did not quite reach an equilibrium swollen state. The radius of the calcium-depleted capsid can be extrapolated to an “equilibrium radius” 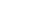 by fitting the 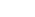 curve (Figure 2) to 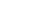 = 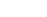
−(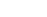
−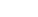)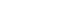. With this fit an equilibrium radius of 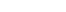 is predicted, about a quarter of a percent higher than the value at the end of the 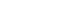 simulation. The weak increase in the radius of the calcium-containing capsids is probably an artifact due to the lack of the RNA molecule and confirms the long-term unstable nature of the RNA-free capsid.

Swollen STNV capsids can be returned to their native radius by lowering the pH [8], suggesting that the mechanism behind the swelling is electrostatic repulsion of charged aspartate and glutamate ligands. A similar effect was deduced from electrostatics calculations of the native and expanded structures of the CCMV capsid [23]. The protonation state of the carboxyl groups is probably coupled to the magnitude of the expansion. The crystals of the expanded STNV formed at pH 6.5 and the expansion was only moderate, so the capsid may have been protonated at one of the calcium binding carboxyl groups. Since the type II site has the highest local density of negative charge, we decided to protonate one of the three carboxyl groups at each type II binding site in our simulations of the calcium-free capsid.

Analytic ultracentrifugation measurements estimate that the STNV particle can expand up to 7% when treated with EDTA [8]. The crystal structure of the expanded virion showed a radial expansion of between 0.1–0.4 nm, equivalent to 1%–5% (higher closer to the icosahedral 3-fold axis). This agrees well with what we observed in the simulations: an average increase in the 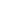 of 2.5% (Table 1) and peak RMSd values of the 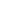 of the shell domain above 0.35 nm at the 2-fold subunit interface, e.g. residues 96 and 122, and at the 3-fold axis, e.g. residues 25 and 195 (Figure 5A).

### Permeability coefficients

The osmotic water permeability 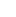 of the capsid is comparable to lipid membranes, which usually have 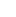 = 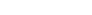 cm/s [24]. The protein shell is thinnest at the 5-fold axis, but the calcium ion at the type III site is the one that is most difficult to chelate with EDTA [25], something which was corroborated by spectroscopic measurements showing that the type III site has a remarkably high binding affinity to the 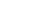 analog 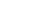 (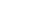 nM) [26]. In the simulations of the capsid without calcium, the coordinating cage of oxygen ligands was best preserved at the type III site. Sodium ions or water molecules replaced the divalent ion on the icosahedral 5-fold axis and obstructed the opening, resulting in minimal permeability even without 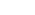. Instead, we observed extensive water permeation on the 3-fold axis and in a region close to the 3-fold axis between the 3-fold and the 2-fold axis (Figure 4). The cluster of four calcium binding sites around the 3-fold axis contains several carboxyl groups from aspartate and glutamate residues. Removing the calcium ions introduces a large amount of net negative charge that caused the subunits to move apart, creating water pockets at the 3-fold axes causing increased water permeability. If the entire region around the 3-fold axis is considered a water pore, the permeability of it is comparable to that of membrane proteins that function as water pores, e.g. mammalian aquaporins that have reported single pore permeability of 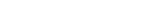 [27].

### Comparison to the expanded structure of TBSV

The difference in size and triangulation number makes it difficult to compare the expanded structure of TBSV and STNV. The capsid of TBSV consists of 180 identical subunits in a T = 3 arrangement where each of the 60 icosahedral asymmetric units consists of three proteins in slightly different configuration. At the center of these three proteins, there is a so called quasi-3-fold axis that relates three approximately equivalent protein positions [28]. The six calcium binding sites of TBSV are located pairwise between pairs of subunits in the asymmetric unit and each site has five acidic residues from both proteins. The swollen TBSV was crystalized at pH 7.5 and the structure is about 7% larger than the native one. The most predominant structural change is that large openings appear between the quasi-3-fold and 2-fold related subunits [10]. The 3-fold axis of STNV resembles the quasi-3-fold axis of TBSV. The interfaces between the proteins around these axes contain six (TBSV) respectively four (STNV) 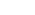 binding sites (Figure 1), coordinated by carboxylic groups. In both capsids the largest structural differences between the expanded and native structures can be found here.

### Factors affecting capsid stability

Not all viruses that have calcium binding sites in the capsid show a swelling behavior. The rhinoviruses have a calcium binding site on the 5-fold axis [7], but do not swell upon removal of these ions. Interestingly, this binding site show striking similarities to the type III calcium binding site in STNV. In both cases five backbone carbonyl oxygens point at the 5-fold axis where the ions are bound. The 5-fold axis is a region with high degree of symmetry constraints and putting an ion there solves the problem of fulfilling the symmetry at a very congested interface. We therefore propose that the carbonyl type of binding sites have a more structural role, while the carboxyl type of binding sites may play a role in the dissolution of the capsid upon infection. This would explain the low degree of structural change at the 5-five fold site in our simulation and their high propensity to bind other cations or water molecules, and this is in line with the relatively higher affinity for ions at these sites [25], [26].

There are no atomic structures of the STNV genome available, although a recently published high-resolution structure shows short fragments of RNA [14]. RNA-free virus-like particles of the STNV coat protein have not been observed so far, but the coat proteins of similar viruses readily form empty shells [29], [30]. Rather than inserting a modeled RNA structure into the capsid we decided to perform the simulations without RNA. The fact that RNA nevertheless may contribute to the stability of the capsid is evidenced from our simulations at physiological salt concentration, providing more shielding of electrostatic interactions. The radial expansion is less pronounced in this case (Figure 2) and the number of protein-protein hydrogen bonds seems to increase slightly (Table 1), a typical “salting out” effect.

Previous all-atom molecular dynamics simulations of capsids and virus-like particles focused on specific properties like the mechanical strength using force probes [17], [18] or the effect of hydration on coherent diffraction from single virus particles [31]. It has become clear though that virus simulations are sensitive to the starting conditions, like a balanced amount of water on the inside, and they require long equilibration times [18], [32]. Therefore we paid special attention to the preparation of the starting structures with an extensive equilibration protocol with part of the counter ions specifically added to the interior cavity to avoid a sudden influx of solvent ions that could disrupt the protein-protein interactions and by carefully balancing of the hydrostatic pressure inside and outside of the capsid. By doing control simulations with 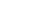 and be varying the ionic strength we can draw firm conclusions on the effect of 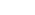 removal on STNV.

### Conclusions

The simulations presented here illustrate the mechanism by which an entire virus capsid can transform from a closed configuration into an open one with significantly increased water permeability. The magnitude of the expansion in the simulations is in good agreement with experiments. The higher flexibility and the degradation of the icosahedral symmetry in combination explain why it has been difficult to crystallize expanded capsids. Our work strongly suggests that there are two types of calcium binding sites, playing different roles in the virus lifecycle. The binding site on the 5-fold axis has a more structural role and is less involved in the capsid expansion. The binding sites between 2- and 3-fold related subunits contain many charged carboxylic side chains. Dissolution of the capsid is initiated here due to the electrostatic repulsion between these residues, if the 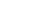 ions are removed.

## Materials and Methods

All preparations, simulations and analyses were performed with the GROMACS simulation package version 4.5.3 [33] unless otherwise stated.

### System preparation

The starting structures were prepared from the X-ray crystal structure of the coat protein of STNV (PDB ID: 2buk) [2]. Since residue 1–11 can not be resolved in the crystal structure, these were modeled as an 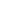-helix in a direction that did not cause steric clashes with neighboring proteins. The full capsid was generated by applying the icosahedral rotation-translation matrices in the PDB file. One 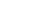 ion was kept at each of the 92 calcium binding sites. The Amber99sb-ILDN forcefield [34], [35] was used for the protein combined with TIP3P water [36] in a rhombic dodecahedron simulation box with a side of about 25 nm. Water molecules on the inside of the capsid were replaced with 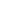 ions to obtain a neutral system (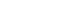). Additional 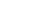 and 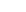 ions were added to obtain a system with an approximately physiological ion concentration of 150 mM (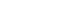). The calcium ions were removed, a proton was added to one Asp55 side chain at each of the type II calcium bindings sites and the number of counter ions was adjusted to obtain two neutral calcium-free systems (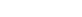 and 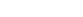 respectively). Each system consisted of roughly 1.2 million atoms (Table 1).

### Preparatory simulations

The starting structures were energy minimized and subsequently the solvent was equilibrated for 10 ns while restraining the protein atoms and the calcium ions to the crystal coordinates with harmonic potentials with a force constant of 1000 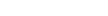. During the equilibration a 2 fs integration timestep was used and the neighbor lists were updated every 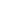 timestep. Short range non-bonded Van der Waals (Lennard-Jones) and Coulomb interactions were calculated within a cut-off radius of 1.15 nm. The long range electrostatic interactions were calculated with the particle mesh Ewald (PME) method [37] with a grid spacing of 0.133 nm. The long range Lennard-Jones interactions were analytically corrected for in the calculation of the pressure and the energy. The pressure of the simulation box was kept at an average of 1 bar using the isotropic Berendsen barostat [38] with a time constant of 25 ps and a compressibility of 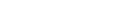. The solvent and the capsid were coupled separately to an external heat bath at 300 K with the velocity-rescaling thermostat [39] using a time constant of 0.5 ps. Water molecules were constrained using the SETTLE algorithm [40] and the covalent bonds in the proteins were constrained using the P-LINCS algorithm [33]. Boundaries were treated periodically.

### Production simulations

After the equilibration, the position restraints were removed and an integration time step of 4 fs was used to generate 1 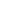 trajectories. In addition to constraining bond lengths, virtual hydrogen atoms were used [41] which allows slightly longer time steps. The isotropic Parrinello-Rahman barostat [42], [43] was used to keep the average pressure at 1 bar with a time constant of 1 ps. The calcium ions were tethered to the oxygen ligands using harmonic potentials with force constants of 5000 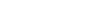. All other simulation parameters were the same as during the equilibration. The trajectories were sampled every 50 ps for analysis. The production simulations were calculated in parallel on a Cray XE6 system over 2016 cores (612 of which for PME calculations) at a speed of 30 ns/day.

### Analysis

Unless otherwise stated, all trajectories were analyzed at 1 ns intervals and final values were calculated as the average over the last 100 ns. Root-mean-square displacement (RMSd) and fluctuation (RMSf) was calculated as:
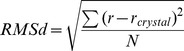
(1)

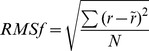
(2)


The number of 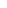-helical residues was calculated using the g_helix program of the GROMACS package. In the water permeability analysis water molecules were counted when they had traversed the entire width of the capsid shell. Visual inspection did not imply a single-file type of permeation mechanism, which justifies that the distinction between a diffusive and osmotic permeation coefficients was not required [21]. The osmotic water permeability coefficient, 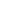, and the single pore osmotic water permeability coefficient, 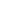, was calculated as the average permeability in both directions using these formulas:
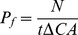
(3)

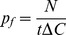
(4)


Where N is the number of permeation events, t is the duration, A is the area and 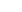 is the concentration gradient, i.e. 55 mol/L for pure water. The capsid was approximated to have the same area as a sphere with radius 8 nm.
